# Characterisation of chronic obstructive pulmonary disease (COPD) in never-smokers and ever-smokers from a population-based cohort

**DOI:** 10.1136/bmjresp-2025-003578

**Published:** 2026-02-27

**Authors:** Pernilla Sönnerfors, Petra Kristina Jacobson, Anders Andersson, Leif Hilding Bjermer, Anders Blomberg, Heléne Blomqvist, Jonas S Erjefält, Iryna Kolosenko, Andrei Malinovschi, Terezia Pincikova, Ellen Tufvesson, Åsa M Wheelock, Christer Janson, Hans Lennart Persson, Magnus Sköld

**Affiliations:** 1 Division of Immunology and Respiratory Medicine, Department of Medicine Solna, Karolinska Institutet, Stockholm, Sweden; 2 Medical Unit Occupational Therapy and Physiotherapy, Women's Health and Allied Health Professionals Theme, Karolinska University Hospital, Stockholm, Sweden; 3 Department of Respiratory Medicine in Linköping, Department of Health, Medicine and Caring Sciences, Linköping University, Linköping, Sweden; 4 COPD Center, Department of Respiratory Medicine and Allergology, Sahlgrenska University Hospital, Gothenburg, Sweden; 5 COPD Center Department of Internal Medicine and Clinical Nutrition, Institute of Medicine, Sahlgrenska Academy, University of Gothenburg, Gothenburg, Sweden; 6 Department of Clinical Sciences, Respiratory Medicine, Allergology and Palliative Medicine, Lund University, Lund, Sweden; 7 Department of Public Health and Clinical Medicine, Umeå University, Umeå, Sweden; 8 Department of Respiratory Medicine and Allergy, and Center for Molecular Medicine, Karolinska University Hospital, Stockholm, Sweden; 9 Unit of Airway Inflammation, Department of Experimental Medicine Sciences, Lund University, Lund, Sweden; 10 Department of Medical Sciences, Clinical Physiology, Uppsala University, Uppsala, Sweden; 11 Stockholm CF Center, Albatross, Karolinska University Hospital, Huddinge, Sweden; 12 Department of Medical Sciences, Respiratory, Allergy and Sleep Research, Uppsala University, Uppsala, Sweden

**Keywords:** COPD epidemiology, Respiratory Measurement, Respiratory Function Test, Pulmonary Disease, Chronic Obstructive, Physical Examination, Emphysema

## Abstract

**Background:**

Chronic obstructive pulmonary disease (COPD) in never-smokers may have other clinical characteristics than tobacco smoking-related COPD.

**Research question:**

What are the risk factors, biomarkers, respiratory symptoms and health status in never-smoking individuals with COPD?

**Study design and methods:**

We investigated never-smokers with COPD (n=154, mean age 60 years) from the population-based Swedish CArdioPulmonary bioImage Study (SCAPIS), and compared them with four control groups: never-smokers with normal lung function (n=281), current smokers with normal lung function (n=97), ex-smokers with COPD (n=103) and current smokers with COPD (n=55). COPD was defined as forced expiratory volume in 1 s (FEV_1_)/forced vital capacity (FVC) less than the lower limit of normal (LNN) after bronchodilation. We examined fractional exhaled nitric oxide (FeNO), blood biomarkers, respiratory symptoms, health status, medical history and living conditions.

**Results:**

The never-smoker COPD group reported more respiratory symptoms and worse health status than never-smokers with normal lung function, but fewer symptoms, milder airflow limitation and better health status compared with ex-smokers and smokers with COPD. Never-smokers with COPD had more self-reported asthma. Moreover, never-smokers with COPD had higher Immunoglobulin E sensitisations to a mix of aeroallergens, higher geometrical mean FeNO levels and blood eosinophil counts than never-smokers with normal lung function. When participants with self-reported asthma were excluded, never-smokers with COPD still had more wheeze, cough and higher FeNO.

**Conclusion:**

Never-smokers with COPD had more respiratory symptoms and elevated markers of type-2 inflammation, suggesting they might represent a distinct clinical phenotype which may differ from smoking-related COPD. They may therefore need to be treated and followed differently.

**Trial registration number:**

NCT03049202.

**WHAT IS ALREADY KNOWN ON THIS TOPIC**

- Chronic obstructive pulmonary disease (COPD) in never-smokers is common.

**WHAT THIS STUDY ADDS**

- Never-smokers with COPD differ from smoking-associated COPD regarding respiratory symptoms, airway obstruction, comorbidities and biomarkers.

**HOW THIS STUDY MIGHT AFFECT RESEARCH, PRACTICE OR POLICY**

- Never-smokers with COPD may represent a distinct clinical phenotype and may need to be investigated and monitored differently.

## Introduction

 Chronic obstructive pulmonary disease (COPD) is a major public health concern, characterised by chronic airflow limitation and often progressive loss of lung function.^1^ The major risk factor for COPD is tobacco smoking. However, COPD in never-smokers is common, but estimates of its prevalence vary depending on the studied population and sociodemographic factors. Epidemiological studies, of which most have used a COPD definition based on spirometry, have shown that a broad range (22%–51%) of people affected by COPD have never smoked.^2 3^

Reported risk factors for COPD in never-smokers include air pollution, passive tobacco smoking and occupational exposures,^2 4 5^ premature birth, low birth weight,^6 7^ low BMI and increasing age.^45 8^,^10^ In addition, asthma is also a potential risk factor for the development of chronic airflow limitation and COPD.^12 4 8^,^12^

Population-based studies have primarily focused on smoking-related COPD, while the number of large-scale studies addressing COPD among never-smokers is limited.^2 8^ Additionally, never-smokers with COPD have consistently been excluded from clinical trials. Since some clinical features seem to differ from smoking-related COPD, there is a need to increase the knowledge of clinical characteristics, prognosis, causal factors and disease mechanisms in this group, as they might benefit from other treatment strategies than individuals with smoking-related COPD. The Lancet Commission on COPD suggested that COPD be classified into five types according to the known main risk factors: genetically determined, abnormal lung development, infections, smoking or vaping, and environmental exposure.^13^ Based on this suggestion, a new categorisation of COPD has recently been proposed by the Global Initiative for Chronic Obstructive Lung Disease (GOLD), including non-smoking causes.^1^

Given the relatively high prevalence of COPD in never-smokers and the lack of large population-based cohort studies, we aimed to characterise and compare this group with ex-smokers and smokers with COPD, as well as never-smokers and smokers with normal lung function. We hypothesised that never-smokers with COPD comprise a distinct phenotype with different clinical characteristics, risk factors, biomarkers, respiratory symptoms and health status compared with smoking-related COPD.

## Materials and methods

### Study design

We employed a multicentre, case–control study design. Data were collected both from an extensive digital questionnaire (self-reported information) and on site at six University hospital clinics in Sweden during the years 2017–2023. The reporting follows guidelines from the Strengthening the Reporting of Observational Studies in Epidemiology Statement.^14^

#### Participants

The Swedish CArdioPulmonary bioImage Study (SCAPIS) is a multicentre study investigating a population-based sample from the general Swedish population (n=30 154).^15^ From the SCAPIS study office, a list was received of all their eligible self-reported never-smokers with forced expiratory volume in 1 s (FEV_1_)/forced vital capacity (FVC) ratio <0.7 after bronchodilation, and further matching study groups were recruited consecutively. In SCAPIS, the FEV_1_/FVC ratio was presented using the European Community of Coal and Steel (ECCS) standards.^16^ From this cohort, we invited participants aged 50–75 years, based on their smoking history and lung function, to participate in the current study, coined BRONCHO-SCAPIS (Clinical Trial number: https://www.clinicaltrials.gov/study/NCT03049202.^17^ Five groups were recruited: never-smokers with COPD, never-smokers with normal lung function, smokers with normal lung function, ex-smokers with COPD and smokers with COPD (online supplemental figure 1). We re-examined the participants’ lung function and reassessed smoking history by telephone interviews and questionnaires. Participants defined as ‘never-smokers’ had a self-reported smoking history of <100 cigarettes or 20 cigars in their entire lifetime and had not smoked at all during the last 2 years. Participants defined as ‘Ex-smokers’ had ≥10 pack years of smoking and >2 years since smoking cessation. Participants defined as ‘Current smokers’ were still regularly smoking ≥10 cigarettes/day and had been during the last year. Normal lung function was defined as postbronchodilator FEV_1_/FVC of ≥0.70, FEV_1_/FVC z-score of ≥−1.64 (=lower limit of normal, LLN) and a FEV_1_ of ≥90% of predicted value. This threshold of FEV_1_ >90% of predicted value was implemented to create a clearer distinction between study groups and to minimise overlapping classifications. COPD was solely based on post-bronchodilator spirometry value of: FEV_1_/FVC of <0.70, FEV_1_/FVC z-score of <−1.64 and a FEV_1_ of 50–100% of predicted value. The LLN of the FEV_1_/FVC z-score was used to ensure that participants categorised into the group with COPD had a chronic airflow obstruction. No criteria regarding self-reported respiratory symptoms were used for the categorisation of COPD. (online supplemental figure 1) Participants were rescheduled for inclusion if they had symptoms of upper airway infection and were then invited one week after complete recovery. None of the participants had signs of exacerbations within the last 3 months.

**Figure 1:**
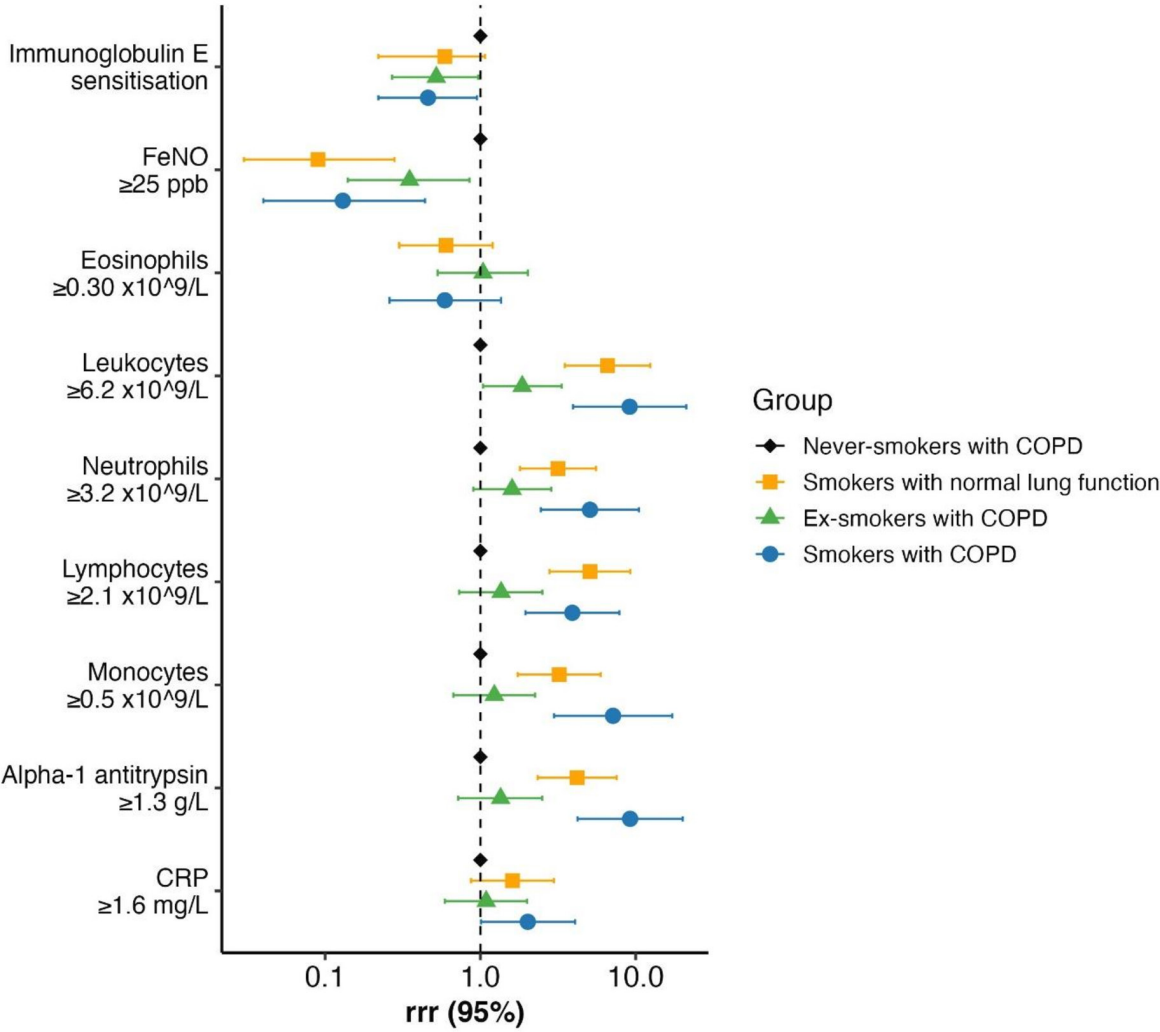
Forest plot of associations and relative risk ratios (RRR) between inflammatory and blood biomarkers in the five study groups. The group of never-smokers with normal lung function was set as the reference group. Relative risk ratios with 95% CIs are given. COPD, chronic obstructive pulmonary disease; CRP, C reactive protein; FeNO, fractional exhaled nitric oxide.

#### Questionnaires

The modified Medical Research Council dyspnoea scale (mMRC)^18^ was used to evaluate dyspnoea. The COPD Assessment Test (CAT)^19^ and the St George’s Respiratory Questionnaire (SGRQ),^20^ which both measure the distinct characteristics of health in patients with COPD, were also employed. In addition, an extensive digital questionnaire was used to include detailed background information, addressing living conditions, education, childhood and upbringing, respiratory symptoms, allergies, asthma, comorbidities, medications, tobacco use, physical activity and occupational exposures (see online supplemental table 1). Questions on allergies covered pets, pollen, food allergies, eczema and eye and nose symptoms. Questions on asthma included whether they had been diagnosed by a physician, age at first asthma attack and age at diagnosis. In the section focusing on respiratory symptoms, participants provided information on cough, breathlessness, wheezing and breathing difficulties. Physical activity and exercise training were reported in hours per week and at different levels.

**Table 1:** Clinical characteristics of the 690 included participants, divided into five study groups

	Never-smokers with COPD (n=154)	Never-smokers with normal lung function (n=281)	Smokers with normal lung function (n=97)	Ex-smokers with COPD (n=103)	Smokers with COPD (n=55)	P value
Age (years), mean±SD	60±5	60±5	62±5	64±4	62±4	<0.001
Range (min-max)	(52–73)	(52–70)	(51–73)	(54–72)	(56–70)	
Women, n (%)	56 (37)	124 (44)	52 (52)	62 (60)	39 (53)	<0.01
SaO_2_ (%), mean±SD	97±2	98±2	97±2	97±2	96±2	<0.001
BMI, mean±SD	26.8±4.8	26.1±4.1	26.3±4.6	28.1±4.3	26.4±4.8	<0.01
Dynamic spirometry, mean±SD						
FVC (% pred.) post BD	111±16	117±15	116±12	111±16	114±14	<0.001
FEV_1_ (% pred.) post BD	87±12	110±13	109±12	78±12	79±13	<0.001
FEV_1_/FVC post BD	0.61±0.05	0.77±0.05	0.77±0.04	0.58±0.07	0.57±0.08	<0.001
BDR FEV_1_ (%)	9.2±9.1	3.3±4.1	2.6±4.2	10.3±9.0	7.9±7.9	<0.001
Smoking n (%)						
Mother smoked, n (%)	49 (32)	81 (29)	43 (45)	48 (47)	24 (44)	0.01
Father smoked, n (%)	75 (49)	128 (46)	58 (61)	63 (61)	39 (72)	<0.0001
Pack-year, mean±SD	n.a.	n.a.	32±14	26±12	39±13	<0.0001
Passive smoking, n (%)	88 (58)	152 (55)	55 (59)	49 (49)	30 (58)	0.606
Self-reported comorbidities, n (%)						
Parental allergy	44 (29)	57 (20)	23 (23)	29 (28)	15 (28)	0.26
Allergic rhinitis	70 (46)	91 (33)	28 (29)	41 (40)	12 (22)	<0.01
Asthma	61 (40)	32 (12)	12 (12)	25 (25)	7 (13)	<0.001
Pet allergy	48 (32)	45 (16)	11 (11)	17 (16)	3 (6)	<0.001
Pollen allergy	56 (37)	69 (25)	15 (16)	20 (19)	9 (17)	<0.01
Food allergy	21 (14)	37 (13)	3 (3)	9 (9)	9 (17)	<0.05
Eczema	31 (20)	66 (24)	25 (27)	32 (31)	11 (20)	0.29
Worsening of respiratory symptoms/exacerbation	27 (18)	29 (11)	7 (7)	28 (27)	11 (20)	<0.0001
Myocardial infarction	1 (1)	3 (1)	4 (4)	6 (6)	2 (4)	0.007
Heart failure	0	1 (0.4)	1 (1)	1 (1)	1 (2)	0.09
Atrial fibrillation	4 (3)	3 (7)	2 (2)	3 (3)	2 (4)	0.62
Stroke	1 (1)	5 (2)	2 (2)	6 (6)	2 (4)	0.30
Hypertension	49 (32)	79 (29)	35 (37)	50 (49)	23 (43)	0.001
Diabetes	6 (4)	12 (4)	11 (12)	9 (9)	6 (11)	0.003
Obstructive sleep apnoea	15 (10)	26 (9)	13 (14)	6 (6)	5 (9)	0.39
Depression	20 (13)	26 (9)	29 (31)	16 (16)	8 (15)	<0.0001
Medications, n (%)						
SABA	24 (16)	9 (3)	4 (4)	19 (18)	11 (20)	<0.01
SAMA	0 (0)	0 (0)	0 (0)	0 (0)	0 (0)	n.a
LABA	41 (27)	14 (5)	3 (3)	37 (36)	12 (22)	<0.01
LAMA	9 (6)	3 (1)	4 (4)	27 (26)	12 (22)	<0.01
ICS	40 (26)	15 (5)	2 (2)	27 (26)	9 (16)	<0.01
Anti-leukotrienes	8 (5)	2 (1)	0 (0)	3 (3)	1 (2)	0.013
Phosphodiesterase 4 (PDE4) inhibitor	0 (0)	0 (0)	0 (0)	1 (1)	0 (0)	n.a

Data are presented as mean (±SD) or numbers (%). P value: ANOVA between all groups.

ANOVA, analysis of variance; BD, bronchodilator; BDR, bronchodilator responsiveness (BDR=FEV1 post- FEV1 pre / (FEV1 predicted value) x 100); BMI, body mass index; COPD, chronic obstructive pulmonary disease; FEV_1,_ forced Expiratory volume in 1 s; FVC, forced vital capacity; ICS, inhaled corticosteroids; LABA, long-acting beta 2 agonists; LAMA, long-acting muscarinic antagonists; n, numbers; n.a, not applicable; % pred, % of predicted normal; SABA, short-acting beta 2 agonists; SAMA, short acting muscarinic antagonist; SaO_2_, oxygen saturation.

#### Spirometry and fractional exhaled nitric oxide

A dynamic spirometry test (Jaeger Masterscope, Jaeger Masterscreen, Medikro or Welch-Allyn) included a bronchodilator test (BD response=FEV_1_ post–FEV_1_ pre/(FEV_1_ pre)× 100), which consisted of administering four doses of salbutamol (0.1 mg/dose) inhaled via a spacer. Each participant underwent at least three forced expiratory manoeuvres, producing curves that were reproducible within a 150 mL range, according to American Thoracic Society (ATS) and European Respiratory Society (ERS) technical standards for conducting spirometry.^21^ All participants were seated during the tests, wore a nose clip and received guidance from a respiratory-skilled research nurse or biomedical scientist. The spirometry results were expressed as % predicted using the ECCS^16^ reference values for FEV_1_ and FVC. However, the Global Lung Initiative (GLI) reference values were used for defining obstruction by z-score of FEV_1_/FVC, as previously described.^17^ A fractional exhaled nitric oxide (FeNO) test (EcoMedics CLD88 or NIOX Vero) was also conducted at some sites. This test included at least one correctly performed exhalation at 50 mL/s with the subject in a sitting position.

#### Blood tests and vital signs

Blood tests included measurements of alpha-1 antitrypsin, haemoglobin, blood leucocyte and differential cell counts, C- reactive protein (CRP) and screening for Immunoglobulin E (IgE) sensitisation against a mix of common airborne allergen (birch, timothy, mugwort, mites, cat, dog, horse, mould) test (Phadiatop, Thermo Fisher Scientific Immunodiagnostics, Uppsala, Sweden). Blood oxygen saturation (SaO_2_), heart rate frequency, blood pressure, height and weight were also recorded. BMI was calculated.^22^

#### Categorisation of respiratory symptoms

In order to further evaluate participants’ respiratory symptoms, we categorised them as being ‘with respiratory symptoms’ or ‘with no respiratory symptoms’ to enable a subgroup analysis. Participants ‘with respiratory symptoms’ had scores ≥2 points on the mMRC dyspnoea scale, or ≥10 points on CAT or ≥25 on the SGRQ symptoms domain.

### Statistical analysis

The Statistical Package for the Social Sciences (SPSS, IBM, USA) and the Stata software V.17 (Stata Corp, USA) were used for the analyses. The clinical characteristics were summarised, using descriptive statistics, presented as numbers (n), per cent (%), mean and SD or median and IQR.

χ² test and analysis of variance were used in the bivariate analyses. Multinomial logistic regression was used in the multivariable analyses when comparing the groups regarding demographics, lung function, allergy status and biomarkers after adjustment for age, sex and BMI. Binomial logistic regression was used when comparing the independent association between respiratory symptoms and dyspnoea between groups. Multiple linear and logistic regression was used to study the association between health status (SGRQ and CAT) and the study groups. Adjustments were made for age, sex and BMI. In the blood samples analysis, values below the detection value were recorded as the detection value divided by two. The statistical significance level was set at ≤0.05.

Sensitivity analyses were conducted with group recategorisation in two ways: (A) defining chronic airflow obstruction only using FEV_1_/FVC<0.70, rather than the combination of the fixed ratio and LLN. (B) Calculation of FEV_1_ as percentage of predicted using GLI instead of ECCS.

### Patient and public involvement

There was no patient or public involvement in the planning of the study.

## Results

### Clinical characteristics

A total of 690 participants were included, 154 never-smokers with COPD, 281 never-smokers with normal lung function, 97 smokers with normal lung function, 103 ex-smokers with COPD and 55 smokers with COPD (table 1). Never-smokers with COPD had a mean age of 59.5 years, and 37% were women. Ex-smokers with COPD were older compared with the other groups. Ex-smokers and smokers with COPD had higher BMI. Never-smokers with COPD were less obstructive compared with ex-smokers and smokers with COPD and reported more respiratory comorbidities, such as asthma, allergic rhinitis and allergies (pollen and pets), compared with all other groups. Never-smokers with COPD also had lower exposure to parental smoking and had fewer non-respiratory comorbidities, such as cardiovascular disease, hypertension, diabetes and depression, compared with the groups with a smoking history. However, no differences were observed across the groups regarding parental allergies or eczema. The study groups with COPD used inhaled bronchodilators and corticosteroids to a higher extent and had more episodes of respiratory worsening and exacerbations than the groups with normal lung function. The never-smokers with COPD had respiratory medical treatment to the same extent as ever-smoker COPD. One participant from the group of never-smokers with COPD was excluded due to the inclusion criteria, FEV_1_ <50% of predicted, as were four of the controls who were ever-smokers with COPD. Eighty-eight never-smokers with COPD, and six participants of the controls being ex-smokers, were excluded due to FEV_1_ >100% of predicted, criterion alone.

Participants with a history of smoking were more likely to have non-respiratory comorbidities, such as myocardial infarction, heart failure, hypertension, diabetes and depression. In contrast, never-smokers with COPD had more respiratory comorbidities such as asthma and allergy, compared with all other groups. Never-smokers and ex-smokers had more exacerbations compared with both groups of smokers. The findings remained consistent when adjusted for sex, age and BMI (online supplemental table 2).

### Risk factors and exposures

Never-smokers with COPD exhibited similar physical activity and exercise training patterns as never-smokers with normal lung function and ex-smokers with COPD. (online supplemental figure 2) Among never-smokers, 60%–63% reported a low physical activity level (0–2 hours per week), while among ex-smokers or current smokers, the percentage was higher, 76%–82%. Moreover, only 37% of never-smokers with COPD either did not exercise at all or exercised only monthly, compared with 80% of smokers with COPD and 76% of smokers with normal lung function.

**Figure 2:**
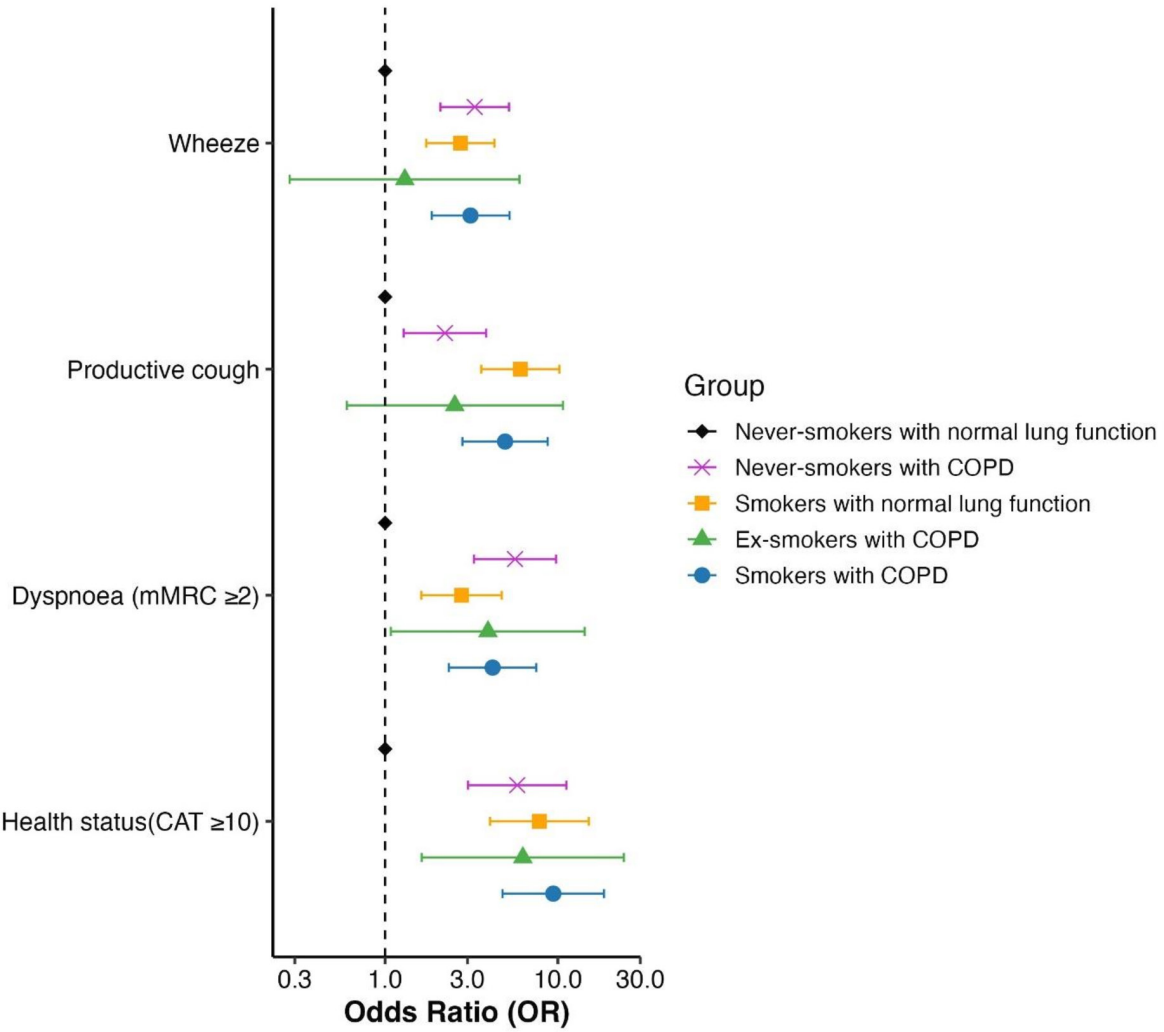
Forest plot of associations of wheeze, productive cough, dyspnoea (mMRC) and health status (CAT), in the study groups. The group of never-smokers with normal lung function was set as the reference group. CAT, COPD Assessment Test; COPD, chronic obstructive pulmonary disease; mMRC, modified Medical Research Council.

No differences were found regarding risk factors and exposures when never-smokers with COPD were compared with never-smokers with normal lung function. Furthermore, smokers and ex-smokers with or without COPD were less likely to be living in villas and were more likely to have smoking parents and lower education levels (online supplemental table 3).

### Biomarkers

Never-smokers with COPD were more frequently sensitised to airborne allergens and had higher FeNO levels compared with all other groups (table 2).

**Table 2:** Inflammatory and blood biomarkers from the 690 included participants divided into the five study groups

	Never smokers with COPD (n=154)	Never smokers with normal lung function (n=281)	Smokers with normal lung function (n=97)	Ex-smokers with COPD (n=103)	Smokers with COPD (n=55)	P value
IgE sensitisation	63 (43)	77 (30)	26 (29)	23 (23)	13 (24)	<0.01
FeNO ppb*	22 (19–27)	19 (17–21)	10 (8–12)	17 (14–21)	8 (6–10)	<0.001
Blood-eosinophils ×10^9^/L	0.15 (0.14–0.17)	0.13 (0.12–0.14)	0.15 (0.13–0.17)	0.15 (0.13–0.17)	0.14 (0.12–0.16)	0.04
Blood-leucocytes ×10^9^/L	5.8 (5.5–6.0)	5.4 (5.2–5.6)	7.5 (7.1.7.9)	6.4 (6.1–6.7)	7.9 (7.4–8.4)	<0.001
Blood-neutrophils ×10^9^/L	3.2 (3.0–3.4)	2.9 (2.8–3.1)	4.2 (3.9–4.5)	3.6 (3.4–3.9)	4.6 (4.3–5.1)	<0.001
Blood-lymphocytes ×10^9^/L	1.8 (1.7–1.9)	1.7 (1.7–1.8)	2.3 (2.1–2.5)	1.9 (1.8–2.0)	2.2 (2.0–2.4)	<0.001
Blood-monocytes ×10^9^/L	0.45 (0.43–0.48)	0.42 (0.40–0.43)	0.55 (0.52–0.59)	0.48 (0.42–0.55)	0.61 (0.57–0.67)	<0.001
Serum-alpha-1 antitrypsin g/L	1.2 (1.1–1.3)	1.2 (1.2–1.2)	1.4 (1.3–1.4)	1.3 (1.2–1.3)	1.5 (1.4–1.5)	<0.001
Serum-CRP mg/L†	1.1 (0.9–1.3)	0.9 (0.9–1.0)	1.3 (1.0–1.8)	1.3 (1.1–1.7)	2.0 (1.5–3.1)	<0.001

Data are presented as numbers (%) and geometric mean (95% CI). P value: ANOVA between all groups.

* Available from 304 participants.

† Available from 504 participants.

ANOVA, analysis of variance; CI, Confidence Interval; COPD, chronic obstructive pulmonary disease; CRP, C-reactive protein; FeNO, fractional exhaled nitric oxide; IgE, Immunoglobline E.

Never-smokers with COPD had slightly higher eosinophil counts in peripheral blood compared with never-smokers with normal lung function (0.15×10^9^/L vs 0.13×10^9^/L). In contrast, participants with a history of smoking displayed the highest levels of total leukocytes and neutrophils. No significant difference was observed in the mean levels of alpha-1 antitrypsin in serum between never-smokers with and without COPD. To evaluate the impact of smoking on inflammatory and blood biomarkers, we did a multinomial logistic regression analysis with never-smokers with COPD as the reference group. Participants who were smokers had more inflammatory cells compared with ex-smokers. The analysis was adjusted for sex, age and BMI. Associations and relative risk ratios are shown in figure 1.

### Symptoms and health status

Never-smokers with COPD reported more wheeze (44% vs 18%) and productive cough (39% vs 19%) and had a lower health status (CAT≥10: 30% vs 12%, p<0.001 and SGRQ total score: 9 vs 4, p<0.001) compared with never-smokers with normal lung function (table 3).

**Table 3:** Wheeze, cough, dyspnoea and health status shown for the five study groups

	Never-smokers with COPD (n=154)	Never-smokers with normal lung function (n=281)	Smokers with normal lung function (n=97)	Ex-smokers with COPD (n=103)	Smokers with COPD (n=55)	P value
Wheeze last 12 months, n (%)	65 (44)	49 (18)	31 (33)	57 (58)	29 (57)	<0.001
Wheeze and breathless, n (%)	19 (13)	23 (9)	12 (13)	21 (21)	7 (14)	0.03
Wheeze when a cold, n (%)	41 (28)	18 (7)	9 (10)	32 (32)	11 (22)	<0.001
Wheeze without effort or cold, n (%)	44 (30)	27 (10)	23 (26)	26 (27)	23 (45)	<0.001
Productive cough, n (%)	59 (39)	52 (19)	56 (59)	43 (43)	36 (67)	<0.001
Bronchitis, n (%)	37 (24)	32 (12)	15 (16)	19 (20)	12 (24)	<0.01
Chronic bronchitis n (%)	15 (10)	17 (6)	6 (6)	9 (9)	6 (12)	0.45
Dyspnoea, mMRC≥2, n (%)	3 (2)	4 (2)	4 (5)	9 (10)	6 (11)	<0.01
CAT total score, median (IQR)	6 (4–11)	4 (2–7)	8 (5–13)	8 (5–12)	10 (7–19)	<0.001
CAT≥10, n (%)	46 (30)	32 (12)	40 (42)	44 (43)	32 (58)	<0.001
SGRQ total score, median (IQR)	9 (4–17)	4 (2–7)	8 (4–17)	16 (8–24)	14 (7–28)	<0.001
SGRQ symptom, median (IQR)	17 (5–35)	6 (0–17)	22 (11–35)	21 (9–39)	36 (16–52)	<0.001
SGRQ activity, median (IQR)	12 (6–24)	11 (6–12)	12 (6–30)	27 (12–42)	18 (12–35)	<0.001
SGRQ impact, median (IQR)	2 (0–9)	0	0 (0–6)	5 (0–11)	4 (0–22)	<0.001

P value: ANOVA between all groups.

ANOVA, analysis of variance; CAT, COPD Assessment Test; COPD, chronic obstructive pulmonary disease; mMRC, modified Medical Research Council; n, numbers; SGRQ, St George's Respiratory Questionnaire.

The findings were further visualised by a forest plot, which shows that the odds ratios (OR) for wheeze, productive cough and CAT score ≥10 were significantly higher for never-smokers with COPD compared with never-smokers with normal lung function (figure 2). However, they reported less wheeze, productive cough, dyspnoea and better health status compared with ever-smokers with COPD. Among all groups, never-smokers with normal lung function reported the least wheeze, cough, dyspnoea and the best health status (online supplemental table 4 and online supplemental figure 3).

**Figure 3:**
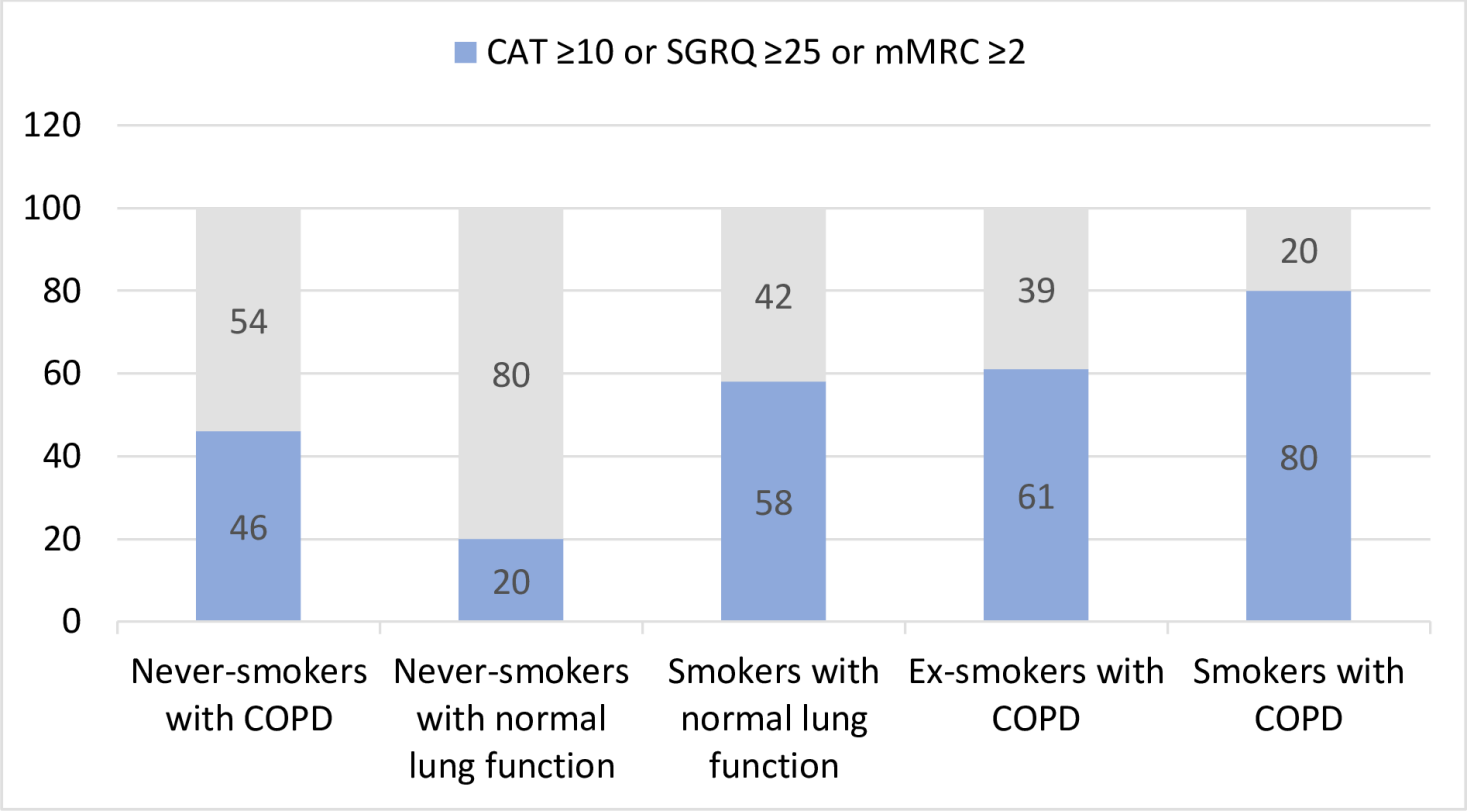
The five study groups were divided into subgroups of those ‘with respiratory symptoms’ and those ‘without respiratory symptoms’. Percentage of the total participants in each group. Four missing data in the group of never-smokers with normal lung function. One missing data in the group of smokers with normal lung function. CAT, COPD Assessment Test; COPD, chronic obstructive pulmonary disease; mMRC, modified Medical Research Council; SGRQ, St George’s Respiratory Questionnaire.

Participants were further categorised into two groups: with respiratory symptoms according to the CAT total score ≥10 or SGRQ symptoms domain ≥25 or mMRC dyspnoea score ≥2, or no respiratory symptoms. Participants from all groups reported respiratory symptoms with variable frequency. Ex-smokers and smokers with COPD had most participants categorised into the subgroup with respiratory symptoms (61% and 80%), but respiratory symptoms were also common among smokers with normal lung function (58%) and, to a certain degree, among never-smokers with COPD (46%). Of note, in the two groups with tobacco smoking-related COPD, 20%–39% reported having no respiratory symptoms (figure 3).

### Impact of self-reported asthma

To evaluate the impact of asthma, we performed a supplementary analysis and excluded participants with self-reported asthma. Never-smokers with COPD without self-reported asthma had more respiratory symptoms, worse health status and increased FeNO levels than never-smokers without COPD. They also showed greater bronchodilator reversibility. However, no significant differences were found in IgE sensitisation, blood eosinophil counts or allergy prevalence compared with never-smokers with COPD without asthma (online supplemental table 4).

### Sensitivity analyses

Defining chronic airflow obstruction based only on FEV_1_/FVC increased the number of never smokers with COPD from 154 to 188 and smokers with COPD from 55 to 61.

Replacing the predicted FEV_1_ values based on ECCS with those based on GLI resulted in the loss of 30 and 14 participants from the groups of never-smokers and smokers with normal lung function, respectively, because of FEV _1_<90% of the predicted. One participant from the group of never-smokers with COPD was also excluded due to FEV _1_>100% of the predicted. The change in group definitions, however, did not change the associations reported above.

## Discussion

This is, to our knowledge, the first cross-sectional study comparing never-smokers with COPD with four relevant control groups. We found that never-smokers with COPD had more productive cough and wheeze, a more pronounced type 2 inflammation and worse health status compared with never-smokers with normal lung function, but fewer symptoms and milder chronic airway obstruction than the groups of ever-smokers. The milder respiratory symptoms in never-smokers with COPD compared with tobacco-related COPD are consistent with findings from a systematic review by Rodríguez García *et al*.^8^

A productive cough was less common in never-smokers with COPD compared with smokers with COPD, consistent with findings from a Chinese population study.^5^ Additionally, wheezing, which is a respiratory symptom identified as one of the most prevalent in never-smokers with COPD,^10^ was common in our cohort.

In our cohort, never-smokers with COPD were more frequently sensitised to airborne allergies and had higher FeNO levels, compared with all other groups. Similar findings were reported in a previous study from New Zealand.^12^ Furthermore, we observed that inflammatory biomarkers such as eosinophil counts in peripheral blood were slightly higher in never-smokers with COPD compared with never-smokers with normal lung function and smokers with COPD. In line with this observation, but in contrast to another study,^23^ we found increased numbers of blood leukocytes in never-smokers with COPD compared with those with normal lung function. Although there was a slight increase in leucocyte counts in never-smokers with COPD compared with those with normal lung function, this difference was modest and less pronounced than the higher levels seen as expected in smokers with COPD, suggesting that leucocyte count may be more influenced by smoking.

We found a higher prevalence of self-reported asthma among never-smokers with COPD compared with never-smokers without COPD, supporting that asthma may be a risk factor for COPD. However, as many as 60% of never-smokers with COPD did not report a previous diagnosis of asthma, which indicates that previous asthma can only partially explain COPD in never-smokers. We cannot exclude that some of the participants categorised as COPD may have asthma with chronic airflow limitation, or an undiagnosed disease. In the subgroup analysis, excluding those with asthma, we observed more wheeze and cough, higher levels of FeNO, but similar blood eosinophil levels. Whether this indicates an ongoing airway inflammation in never-smokers with COPD needs to be further evaluated.

Never-smokers with COPD had a higher level of education, had greater occupational activity, were more likely to live in detached houses rather than apartments and had lower exposure to parental smoking than those with smoking-related COPD. All these parameters may indicate a higher socioeconomic status. Compared with healthy participants, physical inactivity is common in COPD.^24^ People with smoking-related COPD often show a reduced level of physical activity already at the time of diagnosis.^25^ Also, in line with this, smokers and ex-smokers had a higher BMI. We found that never-smokers with COPD reported physical activity levels and physical exercise training patterns similar to never-smokers with normal lung function, also suggesting a higher socioeconomic status and that lifestyle patterns might affect a person’s level of physical activity more than impaired lung function.

Participants in this study were included solely based on spirometry values and smoking history, regardless of any respiratory symptoms. According to GOLD guidelines, respiratory symptoms can be assessed using dyspnoea and health-related quality of life scales.^1^ In the present study, the five study groups were categorised into participants ‘with-’ or ‘without respiratory symptoms’ based on the CAT, mMRC and the SGRQ to enable a subgroup analysis. We found that smoking-related COPD had a higher impact on health status, and this group also reported the most respiratory symptoms. Surprisingly, 20% of never-smokers with normal lung function also reported respiratory symptoms, suggesting that factors other than smoking and airway obstruction may be important in this regard. On the other hand, some ever-smoking participants with COPD did not report any respiratory symptoms at all. These observations challenge the assumption that COPD is always symptomatic^1^ and raise the question of whether a variable as difficult to evaluate as symptoms should be included in the diagnosis of COPD. The use of a restricted range of 50%–100% of the FEV1 per cent of predicted value could have been a limitation of the study. However, in our cohort, only one participant was excluded due to FEV1<50%, in the group of never-smokers with COPD and six in the group of ex-smokers with COPD, we believe that the impact on the generalisability is low.

In all smokers, prioritising smoking cessation interventions is essential, as well as offering annual follow-up by spirometry.^1^

### Strengths and limitations

This study has several strengths. The rigorous selection and categorisation of participants in this study enhances the robustness of our findings. The participants’ smoking history was assessed repeatedly on several occasions, during a telephone interview, in a questionnaire and in person during data collection procedures. This approach was considered to provide as high accuracy as possible. Self-reported smoking status is highly consistent with the urinary cotinine test,^26^ and in-person interviews have been shown to determine smoking status more accurately than solely self-administered questionnaires.^27^ All five study groups were recruited from a large population-based cohort based on their spirometry values and smoking history and underwent a second spirometry.^28^ We used the LLN, defined as the fifth percentile, for the FEV_1_/FVC ratio to identify participants with a chronic airflow obstruction, an approach recommended by the ERS and the ATS.^29^ In a sensitivity analysis, we found that changing the definition of chronic airflow obstruction or the reference values when calculating FEV_1_ as % of predicted had some effect on the number of participants in each category, but did not affect the overall associations. The participants were recruited from different geographic areas with diverse conditions, which may give the study population a higher representativeness of the general population.

However, the study also has limitations, such as a narrow age span. Most participant exclusions were due to discrepancies in lung function levels when recruiting from the SCAPIS cohort. Although this may pose a limitation and introduce potential selection biases, all participants were retested, and their most recent results were used for group categorisation, which likely reduced the impact of such biases.

The inclusion criteria were established to create groups relevant for comparison. In total, only nine participants were excluded due to an FEV_1_<50% of the predicted value, while 94 participants being ever-smokers with COPD were excluded due to having an FEV_1_>100% of the predicted value. These exclusions could potentially influence the generalisability of the findings. Also, the number of participants in each study group may be considered low, but this was due to a strict inclusion strategy based on spirometry parameters, as well as recruitment from an already defined population-based cohort.^15^ However, the small number of participants in each group may have resulted in missing significant differences in the subgroup analysis. A more in-depth characterisation of a possible inflammatory pattern in the airways by bronchoscopy may have revealed novel pathways.

## Conclusions

Never-smokers with COPD reported more respiratory symptoms, had worse health status and more pronounced signs of type 2 inflammation compared with healthy never-smokers. They also differed from smoking-related COPD regarding respiratory symptoms, airway obstruction and biomarkers. Our findings suggest that never-smokers with COPD might represent a distinct clinical phenotype which may need to be handled differently both in the diagnostic and clinical follow-up. However, there is still a lack of clinical trials focusing on this specific group; further research with a longitudinal approach focusing on type-2 inflammation and treatment strategies is needed before any of these results can be implemented into clinical guidelines.

## Supplementary material

10.1136/bmjresp-2025-003578 online supplemental figure 1

10.1136/bmjresp-2025-003578 online supplemental figure 2

10.1136/bmjresp-2025-003578 online supplemental figure 3

10.1136/bmjresp-2025-003578 online supplemental table 1

10.1136/bmjresp-2025-003578 online supplemental table 2

10.1136/bmjresp-2025-003578 online supplemental table 3

10.1136/bmjresp-2025-003578 online supplemental table 4

## Data Availability

Data are available on reasonable request.
