# Supplementary material for: Characterisation of chronic obstructive pulmonary disease (COPD) in never-smokers and ever-smokers from a population-based cohort
Source: BMJ Open Respir Res. 2026 Feb 27;13(1):e003578. doi: 10.1136/bmjresp-2025-003578 (PMC12959065; doi:10.1136/bmjresp-2025-003578)
Supplement: online supplemental figure 1 [file bmjresp-13-1-s001.docx]

**Supplementary Figure 1.**

**Participants included in SCAPIS study, n=30 154**

**Participants received study ID, in BRONCHO-SCAPIS study total, n=1212**

Never-smokers with COPD, **n=524**

Comparison groups, **n=688**

- Never-smokers with normal lung function, n=182

- Smokers with normal lung function, n=181

- Ex-smokers with COPD, n= 215

- Smokers with COPD, n=110

Declined participation or non-eligible, **total, n=114**

- Never-smokers with COPD, **n=31**

- Comparison groups, **n=83**

**Screening procedure at the hospital clinic, total, n=1098**

Never-smokers with COPD, **n=493**

Comparison groups, **n=605**

- Never-smokers with normal lung function, n= 168

- Smokers with normal lung function, n=161

- Ex-smokers with COPD, n= 193

- Smokers with COPD, n=83

Excluded **total, n=408**

Never-smokers with COPD, **n=210**

- Lung function not matching inclusion criteria, or failed spirometry

Comparison groups, **n=198**

- Lung function not matching inclusion criteria, or failed spirometry, n= 197

- Declined, n=1

**Included,** **total, n=690**

Never-smokers with COPD, **n=283**

Comparison groups, **n=407**

**Change of group affiliation**

From never-smokers with COPD to comparison groups, **total, n=129**

In-between comparison groups, **total, n=25**

**Participants included in BRONCHO-SCAPIS study, total, n=690**

Never-smokers with COPD, **n=154**

Comparison groups, **n= 536**

- Never-smokers with normal lung function, n=281

- Smokers with normal lung function, n=97

- Ex-smokers with COPD, n= 103

- Smokers with COPD, n=55

*COPD; chronic obstructive pulmonary disease, SCAPIS; the Swedish CArdioPulmonary bioImage Study*

**Figure 1.** Flowchart of the recruitment process in the present BRONCHO-SCAPIS study.
