# Supplementary material for: Characterisation of chronic obstructive pulmonary disease (COPD) in never-smokers and ever-smokers from a population-based cohort
Source: BMJ Open Respir Res. 2026 Feb 27;13(1):e003578. doi: 10.1136/bmjresp-2025-003578 (PMC12959065; doi:10.1136/bmjresp-2025-003578)
Supplement: online supplemental figure 2 [file bmjresp-13-1-s002.docx]

p<0.001 p-value: ANOVA across all groups

*COPD, Chronic Obstructive Pulmonary Disease*

**Supplemental Figure 2.** Self-reported physical exercise levels presented as monthly, weekly, or no physical exercise training in the five study groups.
