# Supplementary material for: Characterisation of chronic obstructive pulmonary disease (COPD) in never-smokers and ever-smokers from a population-based cohort
Source: BMJ Open Respir Res. 2026 Feb 27;13(1):e003578. doi: 10.1136/bmjresp-2025-003578 (PMC12959065; doi:10.1136/bmjresp-2025-003578)
Supplement: online supplemental figure 3 [file bmjresp-13-1-s003.docx]

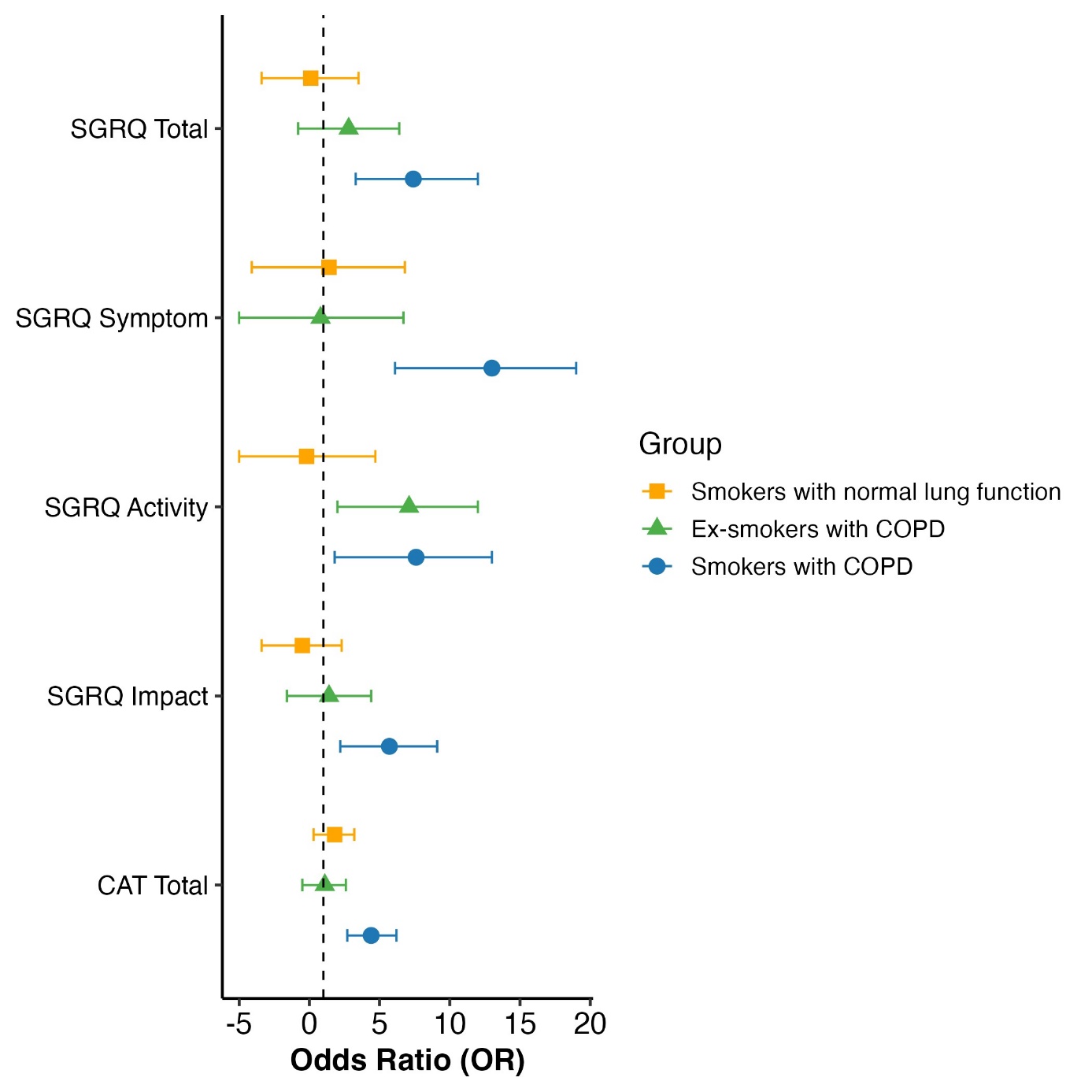


*CAT, COPD Assessment Test; COPD, Chronic Obstructive Pulmonary Disease; SGRQ, St George´s Respiratory Questionnaire.*

**Figure 3.** Forest plot of associations of the S:t George’s Respiratory Questionnaire (SGRQ) and the COPD Assessment Test (CAT) in the study groups, including smokers with normal lung function and those with COPD. The group of never-smokers with COPD was set as the reference group. Odds ratios with 95% confidence intervals are given.
