# Supplementary material for: Characterisation of chronic obstructive pulmonary disease (COPD) in never-smokers and ever-smokers from a population-based cohort
Source: BMJ Open Respir Res. 2026 Feb 27;13(1):e003578. doi: 10.1136/bmjresp-2025-003578 (PMC12959065; doi:10.1136/bmjresp-2025-003578)
Supplement: online supplemental table 1 [file bmjresp-13-1-s004.docx]

**Supplementary Table 1.** Self-reported risk factors in the five study groups.

|  | Never smokers with COPD  (n=153) | Never smokers with normal lung function  (n=281) | Smokers with normal lung function  (n=96) | Ex-smokers with COPD  (n=103) | Smokers with COPD  (n=55) | p-value |
| --- | --- | --- | --- | --- | --- | --- |
| Educational level |  |  |  |  |  | <0.0001 |
| Elementary | 9 (6) | 11 (4) | 19 (20) | 18 (18) | 11 (20) |  |
| High school | 52 (34) | 107 (38) | 44 (47) | 54 (52) | 25 (46) |  |
| College | 92 (60) | 161 (58) | 30 (32) | 31 (30) | 18 (33) |  |
| Occupation |  |  |  |  |  |  |
| Occupational ongoing | 121 (79) | 219 (78) | 59 (62) | 48 (47) | 31 (56) | <0.0001 |
| Living |  |  |  |  |  | <0.0001 |
| Apartment | 62 (41) | 95 (34) | 66 (69) | 72 (70) | 41 (76) |  |
| Detached/semi-detached | 89 (58) | 180 (64) | 29 (31) | 29 (28) | 12 (22) |  |
| Present |  |  |  |  |  | <0.0001 |
| Living: Rural | 14 (9) | 41 (15) | 13 (14) | 13 (13) | 4 (7) |  |
| Small town /suburb | 83 (54) | 152 (54) | 27 (28) | 37 (36) | 19 (35) |  |
| Inner city | 56 (37) | 86 (31) | 55 (58) | 52 (51) | 31 (57) |  |
| Pets | 48 (32) | 83 (30) | 28 (29) | 29 (28) | 17 (31) | 0.98 |
| Pets previously | 109 (71) | 198 (71) | 74 (78) | 80 (78) | 37 (69) | 0.38 |
| Childhood |  |  |  |  |  | 0.001 |
| Living: Rural | 32 (21) | 57 (20) | 19 (20) | 19 (19) | 6 (11) |  |
| Small town /suburb | 89 (58) | 176 (63) | 43 (45) | 55 (54) | 25 (46) |  |
| Inner city | 32 (21) | 47 (17) | 33 (35) | 28 (27) | 23 (43) |  |
| Premature | 13 (8) | 22 (8) | 2 (2) | 13 (13) | 4 (7) | 0.02 |
| Caesarean section | 3 (2) | 6 (2) | 1 (1) | 1 (1) | 1 (2) | 0-50 |
| Pneumonia as child | 11 (7) | 12 (4) | 1 (1) | 6 (6) | 2 (4) | 0.43 |
| Exposures | 51 (34) | 67 (25) | 36 (39) | 36 (35) | 17 (33) | 0.07 |
| Welding | 11 (7) | 30 (11) | 14 (15) | 13 (13) | 7 (13) | 0.40 |
| Rock dust | 12 (8) | 12 (4) | 3 (3) | 5 (5) | 6 (11) | 0.16 |
| Glass wool | 8 (5) | 19 (7) | 13 (14) | 9 (9) | 3 (6) | 0.12 |
| Birds | 2 (1) | 2 (1) | 4 (4) | 3 (3) | 0 | 0.11 |
| Cereals | 7 (5) | 15 (5) | 8 (9) | 4 (4) | 3 (6) | 0.67 |
| Flour | 6 (4) | 7 (3) | 13 (14) | 3 (3) | 4 (7) | <0.0001 |
| Wood dust | 16 (11) | 16 (6) | 17 (18) | 7 (7) | 4 (7) | 0.006 |
| Paper dust | 16 (11) | 14 (5) | 11 (12) | 8 (8) | 6 (11) | 0.14 |
| Textile dust | 7 (5) | 9 (3) | 8 (9) | 9 (9) | 2 (4) | 0.12 |
| Fire smoke | 3 (2) | 8 (3) | 2 (2) | 1 (1) | 0 | 0.62 |
| Irritating gases | 9 (6) | 15 (5) | 3 (3) | 3 (3) | 3 (6) | 0.73 |
| Number of exposures |  |  |  |  |  | 0.04 |
| 0 | 98 (70) | 188 (71) | 47 (52) | 64 (65) | 37 (70) |  |
| 1 | 21 (15) | 44 (17) | 19 (21) | 20 (20) | 6 (11) |  |
| 2 or more | 21 (15) | 34 (13) | 25 (27) | 15 (15) | 10 (19) |  |

Data presented as mean± SD or n (%)

p-value: ANOVA across all groups
