# Supplementary material for: Characterisation of chronic obstructive pulmonary disease (COPD) in never-smokers and ever-smokers from a population-based cohort
Source: BMJ Open Respir Res. 2026 Feb 27;13(1):e003578. doi: 10.1136/bmjresp-2025-003578 (PMC12959065; doi:10.1136/bmjresp-2025-003578)
Supplement: online supplemental table 2 [file bmjresp-13-1-s005.docx]

**Supplemental Table 2.**  Multinominal logistic regression analysis reflecting the confidence interval (95% CI) showing associations regarding clinical characteristics, using never-smokers with normal lung function as the reference group.

|  | Never-smokers with COPD  n=154 | Smokers with normal lung function n=97 | Ex-smokers  with COPD  (n=103) | Smokers with COPD  n=55 |
| --- | --- | --- | --- | --- |
| Age per 10 years | 0.80 (0.52-1.22) | **2.03 (1.23-3.34)** | **7.21 (4.14-12.6)** | **2.49 (1.32-4.68)** |
| Women | 0.77 (0.51-1.17) | 1.31 (0.81-2.13) | **1.92 (1.17-3.16)** | 1.30 (0.71-2.38) |
| *Educational level* |  |  |  |  |
| Elementary school | 1 | 1 | 1 | 1 |
| High school | 0.61 (0.24-1.57) | **0.22 (0.10-0.52)** | **0.30 (0.12-0.74)** | **0.22 (0.08-0.58)** |
| College | 0.81 (0.32-2.04) | **0.09 (0.04-0.21)** | **0.10 (0.12-0.74)** | **0.10 (0.03-0.26)** |
| *BMI groups* |  |  |  |  |
| <20 kg/m^2^ | 1.01 (0.25-4.09) | 2.91 (0.93-9.05) | - | **4.75 (1.39-16.3)** |
| 20-25 kg/m^2^ | 1 | 1 | 1 | 1 |
| >25-30 kg/m^2^ | 1.07 (0.68-1.68) | 1.29 (0.75-2.23) | **1.88 (1.05-3.36)** | 1.32 (0.64-2.70) |
| >30 kg/m^2^ | 1.68 (0.95-2.97) | 1.94 (0.97-3.89) | **5.29 (2.69-10.4)** | **2.86 (1.25-6.58)** |
| *Comorbidities* |  |  |  |  |
| Parental allergy | 1.52 (0.95-2.40) | 1.19 (0.68-2.11) | 1.67 (0.95-2.95) | 1.51 (0.76-2.99) |
| Allergic rhinitis | **1.71 (1.13-2.57)** | 0.80 (0.48-1.35) | 1.24 (0.74-2.05) | 0.51 (0.25-1.04) |
| Asthma | **5.13 (3.11-8.44)** | 1.03 (0.50-2.11) | 1.91 (1.00-3.63) | 1.02 (0.42-2.49) |
| Pet allergy | **2.32 (1.45-3.73)** | 0.67 (0.32-1.36) | 1.04 (0.54-2.02) | **0.29 (0.09-0.99)** |
| Pollen allergy | **1.72 (1.12-2.65)** | 0.55 (0.30-1.04) | 0.74 (0.41-1.36) | 0.57 (0.26-1.24) |
| Worsening of respiratory symptoms/exacerbation | **1.85 (1.04-3.28)** | 0.62 (0.26-1.48**)** | **2.88 (1.52-5.44)** | 1.91 (0.87-4.22) |
| Myocardial infarction | 0.32 (0.05-2.12) | **3.06 (1.32-7.11)** | 2.02 (0.69-5.89) | 1.33 (0.32-5.53) |
| Heart failure | 0.79 (0.23-2.70) | **3.10 (1.40-6.90)** | 1.97 (0.73-5.36) | **2.82 (1.10-16.0)** |
| Hypertension | 0.96 (0.67-1.49) | **1.55 (1.01-2.41)** | **1.57 (1.01-2.46)** | **1.88 (1.10-3.22)** |
| Diabetes | 0.80 (0.30-2.16) | **3.18 (1.45-6.97)** | 1.18 (0.46-3.04) | 2.06 (0.75-5.70) |
| Depression | 0.98 (0.61-1.59) | **2.50 (1.61-3.87)** | 1.13 (0.65-1.95) | 1.06 (0.53-2.10) |

Analysis adjusted for sex, age, and BMI.

*BMI, Body Mass Index; COPD, Chronic Obstructive Pulmonary Disease; CI, confidence interval*
