# Supplementary material for: Characterisation of chronic obstructive pulmonary disease (COPD) in never-smokers and ever-smokers from a population-based cohort
Source: BMJ Open Respir Res. 2026 Feb 27;13(1):e003578. doi: 10.1136/bmjresp-2025-003578 (PMC12959065; doi:10.1136/bmjresp-2025-003578)
Supplement: online supplemental table 3 [file bmjresp-13-1-s006.docx]

**Supplemental Table 3.** Multinominal logistic regression analysis reflecting the confidence interval (95% CI ) showing associations of risk factors and exposures, using never-smokers with normal lung function as the reference group.

|  | Never-smokers with COPD  (n=154) | Smokers with normal lung function  (n=97) | Ex-smokers with COPD  (n=103) | Smokers with COPD  (n=55) |
| --- | --- | --- | --- | --- |
| *Exercise* |  |  |  |  |
| None | 1 | 1 | 1 | 1 |
| Every month | 1.27 (0.66-2.46) | **0.36 (0.19-0.71)** | 0.48 (0.22-1.06) | **0.40 (0.18-0.89)** |
| Every week | 1.08 (0.63-1.85) | **0.12 (0.07-0.22)** | **0.52 (0.29-0.92)** | **0.11 (0.05-0.24)** |
| *Occupation* |  |  |  |  |
| Occupational ongoing | 0.95 (0.55-1.63) | 0.62 (0.35-1.10) | 0.69 (0.39-1.24) | 0.55 (0.27-1.20) |
| *Childhood living* |  |  |  |  |
| Rural | 1 | 1 | 1 | 1 |
| Small town/suburb | 0.88 (0.53-1.47) | 0.80 (0.43-1.50) | 1.16 (0.59-4.02) | 1.54 (0.59-4.02) |
| Inner city | 1.29 (0.69-2.42) | **2.07 (1.03-4.13)** | 1.66 (0.78-3.52) | **4.57 (1.69-12.4)** |
| Mother smoked | 1.12 (0.72-1.73) | **2.26 (1.38-3.71)** | **2.85 (1.70-4.79)** | **2.29 (1.23-4.27)** |
| Father smoked | 1.17 (0.78-1.75) | **1.98 (1.20-3.26)** | **1.95 (1.15-3.30)** | **4.21 (2.00-8.89)** |
| *Present living* |  |  |  |  |
| Rural | 1 | 1 | 1 | 1 |
| Small town/suburb | 1.59 (0.81-3.10) | 0.60 (0.28-1.28) | 0.86 (0.39-1.89) | 1.44 (0.46-4.55) |
| Inner city | 2.01 (1.004.05) | 1.97 (0.96-4.06) | 2.01 (0.92-4.37) | **3.59 (1.17-11.1)** |
| Apartment | 1 | 1 | 1 | 1 |
| Detached/semi-detached | 0.73 (0.48-1.10) | **0.25 (0.15-0.41)** | **0.24 (0.14-0.41)** | **0.17 (0.08-0.34)** |
| *Exposures** |  |  |  |  |
| Exposure to dust and smoke | 1.38 (0.88-2.18) | **2.20 (1.29-3.75)** | **2.23 (1.27-3.91)** | 1.67 (0.84-3.31) |
| Flour | 1.39 (0.45-4.27) | **7.27 (2.70-19.5)** | 1.87 (0.44-8.00) | 3.38 (0.91-12.6) |
| Wood dust | 1.69 (0.80-3.55) | **5.41 (2.48-11.8)** | 2.41 (0.88-6.56) | 1.91 (0.59-6.24) |
| *Number of exposures*** |  |  |  |  |
| 0 | 1 | 1 | 1 | 1 |
| 1 | 0.83 (0.46-1.49) | **2.10 (1.09-4.01)** | 1.60 (0.82-3.14) | 0.83 (0.32-2.15) |
| 2 or more | 1.00 (0.54-1.86) | **4.06 (2.09-7.88)** | **2.38 (1.12-5.08)** | 1.93 (0.82-4.51) |

*COPD, Chronic Obstructive Pulmonary Disease; CI, confidence interval*

Data were adjusted for sex, age, and body mass index.

*Results due to other exposures are presented in Supplemental Table 1.

**Number of reported exposures of the total 11 different exposures (welding, rock dust, glass wool, birds, cereals, flour, wood dust, paper dust, textile dust, fire smoke and irritating gases).
