# Supplementary material for: Characterisation of chronic obstructive pulmonary disease (COPD) in never-smokers and ever-smokers from a population-based cohort
Source: BMJ Open Respir Res. 2026 Feb 27;13(1):e003578. doi: 10.1136/bmjresp-2025-003578 (PMC12959065; doi:10.1136/bmjresp-2025-003578)
Supplement: online supplemental table 4 [file bmjresp-13-1-s007.docx]

|  | Never-smokers with COPD  (n=92) | Never-smokers with normal lung function  (n=245) | p-value |
| --- | --- | --- | --- |
| Age, (years), mean (±SD) | 60±5 | 60±5 | 0.23 |
| Women, n (%) | 29 (32) | 102 (42) | 0.09 |
| BMI, n (%) |  |  |  |
| BMI groups |  |  | 0.17 |
| <20 kg/m^2^ | 2 (2.2) | 6 (2.4) |  |
| 20-25 kg/m^2^ | 35 (38) | 111 (45) |  |
| >25-30 kg/m^2^ | 37 (40) | 102 (42) |  |
| >30 kg/m^2^ | 18 (20) | 26 (11) |  |
| Dynamic spirometry,  mean (±SD) |  |  |  |
| FVC (% pred.) post BD | 112 ±15 | 117±15 | 0.006 |
| FEV_1_ (% pred.) post BD | 86±11 | 110±12 | <0.0001 |
| FEV_1_/FVC post BD | 0.62±0.04 | 0.77±0.05 | <0.0001 |
| BD FEV_1_ rev_._(%) | 7.6±8.0 | 3.3±4.1 | <0.0001 |
| Biomarkers, n (%) |  |  |  |
| Immunoglobulin E | 26 (29) | 63 (28) | 0.80 |
| Eosinophils ≥ 0.30 x 10^9^/L | 15 (17) | 31 (14) | 0.52 |
| FeNO ≥ 25 ppb* | 16 (44) | 20 (24) | 0.03 |
| Self-reported comorbidities, n (%) |  |  |  |
| Parental allergy | 24 (26) | 45 (18) | 0.12 |
| Allergic rhinitis | 27 (30) | 72 (30) | 0.98 |
| Pet allergy | 17 (19) | 33 (14) | 0.24 |
| Pollen allergy | 24 (26) | 54 (22) | 0.43 |
| Food allergy | 8 (8.7) | 27 (11) | 0.53 |
| Eczema | 17 (18) | 52 (21) | 0.57 |
| Myocardial infarction | 0 | 3 (1.2) | 0.29 |
| Heart failure | 0 | 1 (0.4) | 0.68 |
| Atrial fibrillation | 1 (1.1) | 7 (2.9) | 0.35 |
| Stroke | 0 | 3 (1.2) | 0.28 |
| Hypertension | 27 (29) | 64 (27) | 0.75 |
| Diabetes | 5 (5.5) | 10 (4.2) | 0.61 |
| Obstructive sleep apnoea | 10 (11) | 25 (10) | 0.73 |
| Depression | 9 (10) | 19 (8.0) | 0.75 |
| Respiratory symptoms, n (%) |  |  |  |
| Wheeze last 12 months | 21 (24) | 28 (12) | 0.008 |
| Wheeze and breathless | 3 (3.4) | 15 (6.4) | 0.30 |
| Wheeze when a cold | 13 (15) | 10 (4.3) | 0.001 |
| Wheeze without effort or cold | 15 (17) | 17 (7.2) | 0.007 |
| Productive cough | 31 (35) | 36 (15) | <0.0001 |
| Bronchitis | 16 (18) | 18 (7.5) | 0.005 |
| Chronic bronchitis | 7 (7.9) | 10 (4.1) | 0.17 |
| Dyspnoea, n (%) |  |  |  |
| mMRC≥2 | 1 (1.2) | 2 (0.9) | 0.83 |
| Health status, median (IQR) |  |  |  |
| CAT≥10, n (%) | 24 (26) | 20 (8.5) | <0.0001 |
| SGRQ total score, median (IQR) | 6 (4-13) | 4 (2-6) | <0.0001 |
| SGRQ symptom | 12 (0-25) | 6 (0-16) | 0.0004 |
| SGRQ activity | 12 (6-18) | 6 (6-12) | 0.002 |
| SGRQ impact | 0 (0-6) | 0 (0-0) | 0.0002 |

**Supplemental Table 4.** Clinical characteristics in never-smokers with COPD and never-smokers with normal lung function, excluding self-reported asthma
